# Integrative Omics Strategies for Understanding and Combating Brown Planthopper Virulence in Rice Production: A Review

**DOI:** 10.3390/ijms252010981

**Published:** 2024-10-12

**Authors:** Xinfeng Wang, Yaxuan Wang, Houhong Yang, Fang Liu, Yubiao Cai, Jing Xiao, Qiang Fu, Pinjun Wan

**Affiliations:** The National Key Laboratory of Rice Biological Breeding, China National Rice Research Institute, Hangzhou 311401, China; wangxinfeng0211@163.com (X.W.); wangyaxuan604@163.com (Y.W.); 82101221215@caas.cn (H.Y.); qlyj0331@163.com (F.L.); caiyubiao2020@163.com (Y.C.); xiaojing00004@163.com (J.X.)

**Keywords:** brown planthopper, high-throughput sequencing, rice resistance, virulence shift

## Abstract

The brown planthopper (*Nilaparvata lugens*, BPH) is a serious insect pest responsible for causing immense economic losses to rice growers around the globe. The development of high-throughput sequencing technologies has significantly improved the research on this pest, and its genome structure, gene expression profiles, and host–plant interactions are being unveiled. The integration of genomic sequencing, transcriptomics, proteomics, and metabolomics has greatly increased our understanding of the biological characteristics of planthoppers, which will benefit the identification of resistant rice varieties and strategies for their control. Strategies like more optimal genome assembly and single-cell RNA-seq help to update our knowledge of gene control structure and cell type-specific usage, shedding light on how planthoppers adjust as well. However, to date, a comprehensive genome-wide investigation of the genetic interactions and population dynamics of BPHs has yet to be exhaustively performed using these next-generation omics technologies. This review summarizes the recent advances and new perspectives regarding the use of omics data for the BPH, with specific emphasis on the integration of both fields to help develop more sustainable pest management strategies. These findings, in combination with those of post-transcriptional and translational modifications involving non-coding RNAs as well as epigenetic variations, further detail intricate host–brown planthopper interaction dynamics, especially regarding resistant rice varieties. Finally, the symbiogenesis of the symbiotic microbial community in a planthopper can be characterized through metagenomic approaches, and its importance in enhancing virulence traits would offer novel opportunities for plant protection by manipulating host–microbe interactions. The concerted diverse omics approaches collectively identified the holistic and complex mechanisms of virulence variation in BPHs, which enables efficient deployment into rice resistance breeding as well as sustainable pest management.

## 1. Introduction

The brown planthopper (*Nilaparvata lugens* Stål, BPH) is one of the most damaging pests of rice crops and threatens sustained production [1,2]. This insect belongs to the order Hemiptera and family Delphacidae, being highly migratory while dispersing over long distances by air currents [3,4]. In China, BPH outbreaks most often occur in the southern regions as well as the middle and lower reaches of the Yangtze River Basin, where it is more prevalent [5]. Sucking rice juice is one of the behaviors exhibited when BPHs attack, feeding on stems and leaves. Both adults and nymphs congregate at the base of panicles where they insert needle-like mouthparts to feed on plant sap, extracting copious amounts of nutrients, leading to stunted growth or, under severe infestations, death [6,7]. Additionally, female BPHs damage the leaf sheaths and leaves of rice plants during oviposition, piercing rice plant tissues with their probosces to lay eggs, which wounds the host more extensively than direct feeding damage. This wounding increases the lesion size and promotes virus transmission, thereby contributing indirectly but significantly to realized insect-caused losses affecting economic output from fields [8]. Hence, it is very important to know the ecological distribution and damage characteristics of BPHs for a proper control method.

Control measures against the BPH have traditionally relied on chemical insecticides [9]. However, excessive use has led to environmental degradation, harmful pesticide residues affecting human health, and the disruption of natural enemies, which in turn induces insecticide resistance and ecological imbalance [10,11]. Studies have shown that the BPH has developed resistance to various classes of insecticides, further complicating control efforts [12,13,14]. According to the latest data from the Arthropod Pesticide Resistance Database (APRD), the BPH has developed resistance to 36 active ingredients with insecticidal activity, and 492 resistance incidents have been reported [15]. Therefore, employing resistant rice varieties is regarded as the one of most economical and effective control methods [16]. It has been reported that the average number of BPHs on the insect-resistant variety ‘Guiyu 11’ was significantly lower than that on the insect-sensitive control ‘Shayouzhan 202’ in field planting [17]. However, the BPH’s ability to adapt and emerge new virulent biotypes has largely nullified the effectiveness of these resistant varieties, reducing their lifespan [18,19]. For example, the International Rice Research Institute (IRRI) selected and bred the first brown planthopper-resistant rice variety IR26 (containing *Bph1*), followed by the BPH-resistant variety IR36 (containing *Bph2*). However, IR26 lost its resistance after two years, and IR36 resistance was maintained for only eight years [20].

The development of sequencing technologies and related methodologies has generated a wealth of genomic, transcriptomic, proteomic, and metabolomic datasets from insects, improving researchers’ understanding of how they reproduce, develop, and grow [21]. Some studies have identified multiple gene families associated with BPH growth and female fecundity through homologous gene identification and spatiotemporal gene expression analysis, such as the amino acid auxin permease gene (*NlAAAP07*) [22], constitutive heat shock 70 kDa (*NlHSC70-3*, *NlHSC70-4*, and *NlHSC70-5*) [23], and peroxisome biogenesis factor (*NlPEX14*) [24]. The analysis of the genetic information of the BPH has also enhanced our understanding of BPH metabolism, immunity, and biochemical detoxification processes. For instance, one study identified a large number of genes encoding pattern recognition proteins, modulation proteins in the prophenoloxidase (proPO) activating cascade, immune effectors, and molecules involved in immune pathways, such as the Toll, immune deficiency (Imd), and Janus kinase signal transducers and activators of transcription (JAK-STAT) pathways [25]. Other studies have demonstrated the detoxification effects of carboxylesterases (CarEs) [26,27], glutathione S-transferases (GSTs) [28], cytochrome P450 monooxygenase (P450) [29], and uridine diphosphate (UDP)-glycosyltransferases (UGTs) [30] also through omics techniques. These multi-omics findings offer more efficient control methods for integrated pest management.

**Figure 1:**
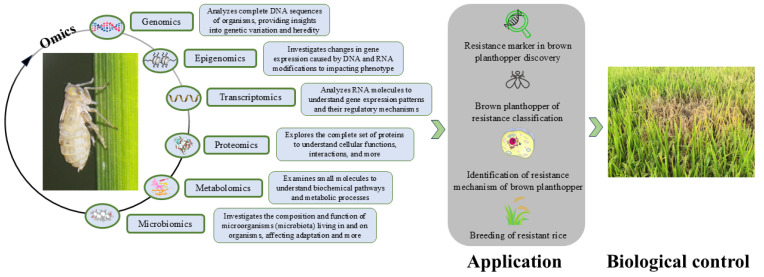
Key tools at the different levels of omics for the harmfulness of brown planthopper discovery.

The dramatic advances in high-throughput sequencing technology in recent years now have great potential for examining the genome, transcriptome, proteome, and metabolome of the BPH. They permit detailed analyses of the genetic structure, functional genes, and regulatory networks of this pest in its interaction with host rice plants (Figure 1). The publication of various versions of the BPH genome, coupled with the comprehensive use of diverse omics technologies, has facilitated major advances in genome assembly, gene expression profiling, resistance gene identification, non-coding RNA functionalities, and the dynamics between virulence-related proteins, metabolites, and symbiotic microorganisms [31]. These results help us understand the genetic evolution of the BPH, the genetic variation characteristics of environmental adaptation, the regulation of growth and development, and the response mechanism to different adversities [32]. This understanding allows us to explore the key targets of BPH prevention and control, improving the efficiency of integrated pest management strategies. This paper reviews the recent advancements in elucidating the virulence mechanisms of the BPH through high-throughput sequencing, with the aim of exploring the multi-omics mechanisms of virulence variation and offering novel insights into and approaches for rice resistance breeding and scientifically informed pest management.

## 2. Genomic Characteristics and Their Applications in Evolutionary Ecology Studies

### 2.1. Genomic Structure and Characteristics of the BPH

Further development of sequencing technologies has eased the assembly process in insect genomes, providing deeper insights into their genomic structure and functional genes, as well as genetic variation [33]. Such an understanding is important for the elucidation of mechanisms of reproduction, environmental adaptability, and insecticide resistance, laying theoretical foundations for the development of more effective control strategies. Multiple versions of the genome of the BPH have already been published due to the continuous improvement of sequencing technologies and the bioinformatics tools used (Table 1). The initial draft genome, put together by scientists from Zhejiang University, had a size of approximately 1140 Mb, an N50 scaffold size of 356.6 kb, a contig N50 size of 24.2 kb, and a GC content of 34.6% using second-generation Illumina technology. It annotated 27,571 protein-coding genes, with a species-specific gene content of up to 59.2%. Abundance of repetitive sequence was revealed in the analysis, with transposable elements (TEs) making up ~38.90% of the genome and different types of repeats [34]. Subsequently, a study determined a de novo genome assembly for the BPH to enhance the genome quality using PacBio and Illumina platforms, increasing the contig N50 to 589.46 kb and scaffold N50s to 77.63 Mb [35]. In parallel, they identified the sex chromosomes for the BPH and two other planthopper species (*Sogatella furcifera* and *Laodelphax striatellus*), revealing high diversity in the gene content among the sex chromosomes of these three species, which suggests the rapid evolution of sex-linked genes. Despite this diversity, high synteny was observed across all chromosomes. The final assembly produced a genome of 1087 Mb with 24,901 annotated protein-coding genes. Another study used Oxford Nanopore sequencing and Hi-C technology to assemble the BPH genome, yielding a chromosome-level assembly of approximately 955 Mb, with a contig N50 length of 1.01 Mb and scaffold N50 of 69.96 Mb. Notably, 95.6% of the assembled scaffolds was anchored onto 16 chromosomes, including 14 autosomes and 2 sex chromosomes (X and Y), filling the previously missing gaps regarding the Y chromosome in hemimetabolous insects [36].

The publication of the BPH genome provides a vital foundation for post-genomic research into its virulence mechanisms, biological characteristics, and resistance mechanisms, facilitating the development of more sustainable and effective pest control strategies. Despite continuous advancements, the chromosome-level genome of the BPH still contains deficiencies, particularly in accurately restoring highly similar DNA sequence fragments to their correct genomic locations due to the abundance of repetitive sequences. This has resulted in many unknown “gap” regions in the reference genome. Recent developments in third-generation sequencing technologies, particularly high-continuity ONT ultra-long and high-accuracy PacBio HiFi sequencing, have made significant strides in achieving telomere-to-telomere assembly, overcoming the challenges of assembling centromeres or highly repetitive regions [37]. Several telomere-to-telomere genomes of crops and insects include rice [38], maize [39], soybean [40], rapeseed [41], and Chinese cabbage [42]. Studies on insects such as the silkworm [43] have been published in 2023, marking a significant advancement. The successful assembly of the BPH’s telomere-to-telomere genome will facilitate detailed studies on high repetitive sequence regions and structural genomic variations in the BPH. This will also aid in a comprehensive understanding of the BPH’s virulence, including insights into newly identified genes, whole-genome methylation, repetitive sequence variations, transposon activity, and other related aspects.

### 2.2. Application of High-Throughput Sequencing in Population Genetics and Evolutionary Ecology Studies of the BPH

Approximately 250,000 years ago, the BPH’s host plant transitioned to rice, evolving into a monophagous herbivore [44]. During their co-evolution, rice developed various resistance mechanisms against this pest. In response, certain BPH populations evolved capabilities to overcome these resistances under selective pressure. Understanding these adaptive evolutionary mechanisms is crucial for breeding resistant rice varieties and managing infestations effectively. The availability of species genomes, combined with significant reductions in sequencing costs and improvements in efficiency, has made whole-genome resequencing a rapid and effective method for studying adaptive evolution and molecular breeding in both plants and animals. Researchers have resequenced 360 samples from 92 BPH populations worldwide. Population genetic analysis revealed substantial genetic differentiation between Australian and Asian populations. Asian populations were further divided into three major groups, East Asia, Southeast Asia, and South Asia, with the South Asian group diverging first. Historical dynamics analysis indicated that the Australian population split from the Asian lineage around 30,000 years ago, expanding until about 2000 years ago. Gene flow analysis showed bidirectional gene flow between East Asia and the Indo-China Peninsula, while migration from Southeast and South Asian populations into the Indo-China Peninsula was predominantly unidirectional [45]. This study not only unveils the migration dynamics, genetic differentiation, and annual influx of Asian BPH populations into major rice-producing regions in China, but also provides a genetic basis for explaining who BPH can rapidly adapt to different rice varieties (virulence variation). The results further deepen our understanding of how insect seasonal migration influences species evolution and population differentiation. Population genetics methods are shown to have great potential for studying the dynamics of wind-borne insect migration. Additionally, exogenous viruses significantly influence host genome evolution by integrating into host genomes [46]. Researchers identified 22 endogenous viral elements (EVEs) from totiviruses in the BPH genome. Association analyses with resequencing data revealed heterogeneity in the EVEs among individual genomes, with some EVEs consistently present, while others appeared selectively, indicating differential evolutionary pressures [47]. This is the first time it has been demonstrated that the domesticated EVEs play host gene functions at the protein level in eukaryotes. It also implies that the current number of known EVEs is seriously underestimated, and they may have contributed significantly to the gene functional diversity of eukaryotes.

In the context of rice–BPH interactions, studies have focused on genomic genetic variation in rice. For example, researchers selected 1520 global rice germplasms for resistance to three BPH biotypes, identifying 3502 single nucleotide polymorphisms (SNPs) and 59 loci associated with BPH resistance in rice [48]. Despite these advancements in BPH genomic research, no similar studies have explored the genetic variation in brown planthoppers in adapting to resistant rice. Adaptive selection may increase the frequency of de novo beneficial mutations or pre-exiting mutations in the population as standing genetic variation [49]. Whole-genome scans of BPH populations may identify new resistance alleles [50]. Future research should analyze SNPs, copy number variations (CNVs), insertions/deletions (InDels), and structural variations (SVs) during BPH adaptation to resistant rice through whole genome sequencing. This would reveal the genetic structure, evolutionary relationships, population history, migration dynamics, and genetic differentiation among various virulence BPH populations from a population genetics perspective.

## 3. Transcriptomic Studies on BPH’s Response to Host Plant Defenses

RNA-seq is a versatile high-throughput sequencing technology that quantifies sequences and quantifies mRNA, small RNA, and non-coding RNA in any cell or tissue [51]. This review section presents how mRNA sequencing technology has been utilized to detect alterations in gene expression profiles in BPHs upon feeding on different types of resistant rice varieties. Furthermore, it investigates the role of non-coding RNAs, such as lncRNAs, circRNAs, and miRNAs, in the fine-tuning of these adaptations to rice resistance (Table 2).

### 3.1. Differential Gene Expression of the BPH to Resistant Rice Varieties

BPHs exhibit varied responses when feeding on different resistant rice varieties. Weakly virulent planthoppers display distinct gene expression patterns in crucial physiological processes, like sugar metabolism, detoxification, cuticle development, molting, and xenobiotic metabolism, when feeding on susceptible rice TN1 versus resistant rice PTB33 [52]. Conversely, highly virulent planthoppers feeding on RH-resistant rice show reduced survival rates, adult female weight, honeydew secretion, egg laying, and fat content, with gene expression alterations primarily in energy and amino acid metabolism, hormone synthesis, and vitamin metabolism pathways [53]. These results indicate that metabolite changes play a key role in BPH adaptation to resistant rice and provide new insights into the metabolic mechanism of pest adaptation to resistant hosts. Additionally, studies suggest that planthopper responses to rice resistance involve mechanisms like starvation stress response, nutrient conversion, oxidative decomposition, and detoxification. For instance, planthoppers on transgenic rice with *Bph6* exhibited upregulated genes linked to starvation, apoptosis, autophagy, reactive oxygen species scavenging, and detoxification within the FoxO signaling pathway [54]. These findings illustrate how different rice varieties can influence planthopper adaptability by altering their energy supply and metabolic processes, enhancing our understanding of how resistant rice varieties thwart planthopper infestations, a critical insight for integrated pest management.

Feeding on resistant rice strains disrupts BPH’s physiological processes, prompting adaptation. Comparative transcriptome analysis of salivary glands from TN1 and Mudgo planthopper populations revealed genes associated with ’digestion and absorption’ linked to virulence [55]. Further research identified differentially expressed genes related to metabolism and immunity in the fat bodies of these populations [56]. Additional studies noted distinct splicing patterns for genes encoding odorant receptors, secreted salivary proteins, and xenobiotic metabolic P450 monooxygenases between Mudgo and TN1 populations [57]. Whole-transcriptome analysis showed that TN1 planthoppers feeding on Mudgo rice significantly downregulated genes involved in cuticle formation, detoxification, metabolite transport, digestion, RNA processing, lipid metabolism, and proteolysis, while insulin signaling genes were upregulated, and glucose and trehalose levels decreased [58]. Research on YHY15 rice, carrying the *Bph15* resistance gene, found upregulated genes related to carbohydrate, amino acid, and nucleotide metabolism, the endocrine system, and signal transduction in avirulent planthoppers [59]. These studies observed the possible role of BPH virulence-related genes by comparing the expression changes in BPH genes with different virulence values. All the results showed that the potential mechanism of BPH virulence was very complex, involving multiple genes, which was helpful to further study the mechanism of BPHs overcoming host resistance.

While mRNA sequencing technology has been instrumental in exploring gene expression patterns in the BPH across different rice varieties, it does not allow for the isolation of specific cell types or individual gene expressions, leading to complex datasets of differentially expressed genes and regulatory networks. Recently, single-cell sequencing technology (scRNA-seq) has been increasingly applied to study cellular heterogeneity, aiming to delineate gene expression at the individual cell level and uncover cell types, subtypes, and states [71]. This approach has revealed that mesophyll, phloem, and xylem cells in resistant rice sheaths confer insect resistance through distinct molecular mechanisms [72]. However, the specific roles of different cell types in BPH virulence variation remain understudied. Advances in single-cell isolation and single-molecule sequencing technologies enable single-cell transcriptome sequencing to identify virulence-related cell types in BPHs. This facilitates the use of RNA interference (RNAi) to target virulence-associated cells or cell subpopulations more accurately, enhancing the efficiency of biological control strategies. Concurrently, single-cell RNA sequencing can be utilized to infer the differentiation trajectories of cell subpopulations associated with BPH virulence, elucidating dynamic changes in gene expression and identifying new targets for developing innovative RNAi-based controls.

### 3.2. Non-Coding RNA Regulation of BPH Response Mechanisms to Feeding on Different Resistant Rice Varieties

RNA-seq has not only highlighted protein-coding messenger RNAs (mRNAs) but also a diverse array of non-coding RNAs (ncRNAs) that are extensively transcribed from the genome [73]. These ncRNAs, which include cis-acting RNAs, trans-acting RNAs, and ligand-binding target proteins, primarily regulate interactions among DNA, RNA, and proteins, thereby initiating subsequent biological processes [74].

Long non-coding RNAs (lncRNAs), which are molecules longer than 200 nucleotides without a protein-coding capacity, are notably diverse and functionally varied in eukaryotes [75]. In a study involving TN1 BPH and those adapted to the resistant rice variety YHY15, researchers identified 3112 lncRNAs and 157 differentially expressed lncRNAs alongside 675 differentially expressed mRNAs. These findings suggest that pathways related to arginine and proline metabolism, glutathione metabolism, and carbon metabolism significantly influence planthopper adaptation to different rice varieties [60]. The study implies a high potential of lncRNAs in the regulation of resistance in BPHs and use for advancing strategies of host defense and high-yield rice breeding.

Moreover, circRNAs (circRNAs) are another type of non-coding RNA belonging to the closed-loop family, lacking 5′ end-cap structures and 3′ polyA tails. They are extensively expressed in eukaryotic transcriptomes with important roles in the process of gene transcription and post-transcriptional translation [76,77]. Changes in the expression of circRNAs may promote the production of new biotypes during the adaption of the BPH to resistant rice. For example, biotype Y planthoppers, virulent to resistant rice YHY15, exhibited higher circRNA expression levels and abundance than those in biotype 1 (susceptible type), among which 19 circRNAs might be involved in the autophagy process [61]. This preliminary finding shows that circRNAs will become part of the mechanisms underlying the adaptive autophagy of planthoppers, extending our general perspective on how non-coding RNA could result in the evolution of enhanced virulence in planthoppers.

MicroRNAs (miRNAs) are small (~22 nt), non-coding RNAs, which act as post-transcriptional regulators of gene expression by pairing with mRNAs of protein-coding genes, culminating in translation repression or mRNA cleavage [78]. Altogether, 41 potential new miRNAs were predicted in TN1 and YHY15 BPH, with a significant differential expression of 26 of them. Most of the differentially expressed miRNAs are related to metabolism pathways, particularly ’glycolysis/gluconeogenesis’ [62]. These provide a foundation for further understanding the role of miRNAs in planthopper adaptation to rice resistance and for the development of novel host defense strategies.

NcRNAs were documented as being involved in the rice-BPH interaction through multi-omics methods; however, to date, most research has not fully explained their molecular and cellular mechanisms. Moreover, RNA epigenetic modifications, such as m6A, m1A, 2′-O-methylation, and 5hmC, can regulate RNA structure and function to affect stability and translation efficiency [79,80]. There have been many studies reporting the role of insect RNA modification in stress, such as m6A levels of *Bombyx mori* being associated with BmNPV infection [81,82], the infection of rice black-streaked dwarf virus (RBSDV) decreasing m6A levels in the midgut cells of *Laodelphax striatellus* [83], the m6A pathway contributing to insecticide thiamethoxam resistance in *Bemisia tabaci* [84], and the m6A pathway also being involved in the host plant adaption of *Plutella xylostella* [85]. However, it has not been reported to date how virulence variation is affected by RNA epigenetic modifications during the feeding process of BPHs on resistant rice materials. The elucidation of dynamic changes in the RNA epigenetic modifications of BPHs enables researchers to thoroughly investigate the epigenetic mechanisms underlying the virulence of this pest. This knowledge facilitates the development of more effective biopesticides by integrating inhibitors such as HDAC inhibitors (HDACis), DNA methyltransferase inhibitors (DNMTis), BET inhibitors (iBETs), and deacetylase inhibitors.

## 4. Proteomics and Metabolomics Responses of BPHs to Resistant Rice

In recent years, the application of post-translational omics technologies, such as proteomics and metabolomics, has advanced significantly for analyzing the complex regulatory and defense systems in pest–host interactions [86,87]. These methods, applied to rice–BPH interactions, can help explore the physiological changes in BPHs when exposed to both resistant and susceptible rice. Key physiological processes responsible for the adaptation of BPHs to rice resistance include apoptosis, lysosomal metabolism, and energy metabolism, which can be identified through the analysis of differentially expressed proteins and metabolites. Moreover, lipidomics research has enhanced our understanding of energy allotment and metabolic adjustments during these interactions, identifying potential virulence markers and adaptation mechanisms, thus contributing to the design of new strategies of biological control strategies [88].

Proteomics, the study of protein arrays and their effects on cells, tissues, and organisms, provides a comprehensive view of gene expression under various environmental conditions. In a study, using iTRAQ tagged with LC-MS/MS, differentially expressed proteins (DEPs) between BPHs feeding on susceptible (TN1) and resistant rice varieties (YHY15), were identified, with 151 upregulated and 107 downregulated. Further analyses showed that the proteins associated with apoptosis and lysosomal metabolism were significantly enriched, indicating their critical role in the adaptation of planthoppers to host defense [63]. These findings reveal how protein contents differ between host-adapted and non-adapted BPH populations enhance our understanding of BPH adaptation at the proteomic level and may also explain traits such as fecundity and survival.

Metabolomics has similarly uncovered distinct metabolic responses to different rice varieties. For instance, planthoppers on resistant rice YHY15 exhibited lower levels of most amino acids but higher levels of urea, succinic acid, and malic acid compared to those feeding on susceptible rice TN1 [64]. Researchers also observed significant metabolic differences in TN1 BPHs when feeding on resistant (YHY15) versus susceptible rice (TN1), indicating nutrient deficiency in the early stages that affects amino acids, fatty acids, and glucose, consequently impacting energy metabolism [65]. Additionally, a study comparing metabolic responses between planthoppers feeding on resistant IR56 and susceptible TN1 rice showed marked decreases in sugars, vitamins, and essential amino acids, while levels of most amides, free fatty acids, and some non-essential amino acids increased in those fed on IR56 [66]. A combined analysis of metabolomics and transcriptomics pinpointed alanine as a key biomarker of BPH adaptation to resistant rice variety IR36, with alanine transfer to pyruvate mediated by ALT playing a crucial role in energy allocation during adaptation [67]. These studies explored metabolic changes in BPHs feeding on different resistant rice varieties, indicating that the insect resistance mechanism of rice affects BPH metabolism in complex ways and highlights the value of metabolomics in understanding intricate plant–insect herbivore interactions.

In line with proteomic and metabolomic studies, lipidomic studies have also revealed ways in which BPHs modulate their lipid profiles upon feeding on different rice cultivars. For instance, an analysis of lipid profiles from the BPHs fed on susceptible rice 9311 and two resistant varieties with genes *Bph6* and *Bph9* by Zheng et al. showed a significantly reduced triglyceride (TG) content, alongside increased phosphatidylcholine (PC) and digalactosyl diacylglycerol (DGDG) levels. This shift from TG to PC and DGDG provides the energy supply required for cell proliferation, maintenance, and growth [68]. This study provides the first detailed description of lipid changes in BPHs fed on resistant and susceptible rice genotypes. Additionally, combining proteomics with other omics approaches can guide microbial metabolic engineering for developing novel biological control agents and promoting innovative control strategies [89,90]. In conclusion, substantial progress in understanding BPH virulence at the protein and metabolomic levels has advanced the development of new biological control methods.

## 5. Diversities of Symbiotic Bacteria in BPHs and Their Role in Virulence

BPHs host a variety of symbiotic bacteria that are highly diverse in species as well as function. These symbionts are closely integrated into the life processes of the planthoppers: growth, development, and reproduction, nutrient metabolism, and immunity [91,92]. Importantly, the pathogenicity of the BPH is strongly associated with the diversity of its symbiotic bacteria [93]. Classic research on BPH symbionts relies heavily on culture-based techniques, for example, fungal isolation from planthoppers’ fat bodies [94]. However, the complex microenvironment in planthoppers often leads to a severe underestimation of symbiont diversity. Following this, the high-throughput sequencing technologies truly revolutionized BPH microbial community research and revealed crucial new insights into the co-evolution mechanisms between planthoppers and their symbiotic microorganisms.

High-throughput sequencing methods opened the way to comprehensive and convenient studies on the structure and composition of insect microbial communities [95,96]. For instance, research into the fat body transcriptomes of BPHs feeding on various resistant rice varieties revealed an abundance of genes involved in variation among the symbiotic bacteria in relation to virulence, including yeast symbionts and Wolbachia [56]. Interestingly, significant changes in bacterial species and abundance of gut symbionts were reported after infestations on different rice varieties. For example, 16S rDNA analysis of the species composition of symbiotic bacteria showed variations among different populations of BPHs, and the cumulative number of symbionts found in populations obtained from planthoppers feeding on resistant varieties like ASD7 and Mudgo is higher than that from those feeding on the susceptible TN1 variety. In the dominant bacterial species as well, there were differences between populations, with Serratia in Mudgo populations, *Aeromonas* in ASD7, and *Arsenophonus* in TN1 [69]. Indeed, the metagenomic comparison showed greater bacterial richness in the resistant populations, with clear differences in the relative abundance of certain bacterial phyla and classes (Firmicutes and Bacteroidetes) and two subdominant classes, *Bacteroidia* and *Clostridia* [70]. These findings demonstrate that BPH gut bacterial communities are highly rich and diverse, and that the host plant variety can significantly influence the structure of these microbial communities. This knowledge enhances our understanding of the interactions between the BPH, its gut microbiota, and host plants, and provides a foundation for future biological control strategies targeting BPHs.

All of these findings are helpful for the development of technologies for microbial pest control, which provides a robust scientific background for studying co-evolutionary dynamics between insect and their symbiotic bacteria [97,98]. Meanwhile, insect microbial associates can influence ecological communities by altering how plants interact with their hosts’ competitors and natural enemies [99]. Manipulating parasitic symbiosis holds great potential for addressing practical challenges in controlling agricultural pests and disease vectors [100]. Thus, the research on the species diversity of symbiotic bacteria in the BPH is of importance for understanding the co-evolution of hosts and probing how the diversity of symbiotic bacteria is related to BPH virulence. The recent advances of high-throughput sequencing technologies allows many symbiotic bacteria to be accurately classified. Still, many scientific questions remain, especially concerning the mechanisms in concrete terms through which symbiotic bacterial diversity impacts virulence and the biological functions of key symbiotic bacteria associated with these traits. Consequently, identifying bacteria strongly associated with BPH virulence and investigating the mechanisms and effects of pest–microbial symbiosis will facilitate the isolation of symbiotic bacteria related to virulence. This approach will aid in targeting microecological controls for BPHs and in developing novel biological bactericides. It will also advance the research into the species diversity, functionality, host interactions, and co-evolution of pest-associated microorganisms.

## 6. Conclusions and Future Perspective

The BPH is known for its high migration ability and reproduction capacity, both of which pose significant challenges in managing virulence variation. Host plant resistance is an effective, environmentally friendly methods for controlling BPHs and maintaining the yield potential of rice cultivars. Future breeding efforts must prioritize the developing cultivars with durable, broad-spectrum resistance. This progress has been made possible due to important advances made in the identification of resistance genes and understanding the interaction mechanism of BPHs with host plants through high-throughput sequencing technologies. A comprehensive application of omics approaches is likely to unravel the complicated mechanisms underlying virulence variation in BPHs, providing a robust foundation for enhancing rice resistance, devising effective pest control strategies, and promoting sustainable rice production.

BPH resistance in rice involves complex regulatory processes in both the insect and the host, making it a multifaceted phenomenon. Therefore, future research must delve deeper into omics studies to better understand BPH–rice interactions and achieve sustainable pest control in rice production. First, high-precision genome assembly and single-cell RNA sequencing are very necessary for deciphering gene structure, functional genes, and the corresponding regulatory networks. This will provide insights into the roles that different cell types play in virulence variation, current cell-specific and dynamic gene expression patterns, and how planthoppers cope with environmental changes. Further investigations into the molecular mechanisms governing the interaction between resistance targets and rice will be essential for identifying new approaches to control BPHs. Second, exploring the specific microbial mechanisms related to BPH resistance and revealing the co-evolutionary processes between symbionts and their hosts will be crucial. Building on this knowledge, specific and efficient microbial agents could offer new avenues for reducing BPH-related damage to rice production. These areas of research are expected to gain increasing attention in rice insect-resistant breeding, ultimately aiding in the control of this destructive rice pest.

## Figures and Tables

**Table 1:** The summary of published *N. lugens* genomes.

Year	Genome Size (Mb)	Sequencing Platform	Chromosome	Scaffold N50	Contig N50	Protein Coding Genes	References
2014	1140	Illumina Hiseq2000	-	356.6 kb	24.2 kb	27,571	[34]
2021	1087	PacBio and Illumina PE150	16	77.63 Mb	589.46 kb	24,901	[35]
2021	955	PacBio and ONT	16	69.96 Mb	1.01 Mb	18,021	[36]

**Table 2:** Multi-omics insights into the virulence of *Nilaparvata lugens*.

Omics	BPH Population	Rice Variety	Key Results	References
Genomics	360 planthoppers from 90 geographical locations worldwide		They clarify the genetic sources of worldwide BPHs and illuminate a landscape of BPH migration showing that East Asian populations perform closed-circuit journeys between Indochina and the Far East.	[45]
Transcriptomics	Susceptible: TN1	Susceptible: TN1 Resistant: PTB33	The 1875 DEGs were identified of which many were annotated to be involved in cuticle development, sugar metabolism, detoxification, molting, and xenobiotics metabolism.	[52]
	Susceptible: Huang Huazhan	Susceptible: TN1 Resistant: RH	The DEGs identified in BPHs feeding on RH were mainly involved in energy metabolism, amino acid metabolism, hormone synthesis, and vitamin metabolism pathways.	[53]
	Susceptible: TN1	Susceptible: TN1 Resistant: Swarnalata	The DEGs related to BPH starvation response (*Nlbmm*), apoptosis and autophagy (caspase 8, ATG13, BNIP3, and IAP), active oxygen elimination (catalase, MSR, and ferritin), and detoxification (GST and CarE) were upregulated in BPHs’ responses to resistant rice.	[54]
	Susceptible: TN1 Resistant: Mudgo		Comparative analysis of the transcriptomes of the two populations revealed that the DEGs related to ‘metabolism’, ‘digestion and absorption’, and ‘salivary secretion’ might be associated with virulence.	[55]
	Susceptible: TN1 Resistant: Mudgo		The DEGs were identified in the fat bodies of the two populations, and these differentially expressed genes related to metabolism and immunity.	[56]
	Susceptible: TN1 Resistant: Mudgo		Genes encoding odorant receptor, secreted saliva protein, and xenobiotic metabolic P450 monooxygenase showed different splicing patterns between Mudgo and TN1 populations.	[57]
	Susceptible: TN1 Resistant: Mudgo	Resistant: Mudgo	Genes involved in cuticle formation, detoxification, metabolite transport, digestion, RNA processing, lipid or fatty acid metabolism, and proteolysis were significantly downregulated during the incompatible interaction, whereas genes involved in insulin signaling were significantly upregulated.	[58]
	Susceptible; TN1 Resistant: YHY15	Resistant: YHY15	Amino acid and nucleotide metabolism, the endocrine system, and signal transduction were upregulated in avirulent BPHs when they fed on YHY15 rice.	[59]
	Susceptible: TN1 Resistant: YHY15		A total of 157 differentially expressed lncRNA was identified. In metabolic-related pathways, arginine and proline metabolism, glutathione metabolism, and carbon metabolism categories were enriched 10 co-expression target genes of these lncRNAs.	[60]
	Susceptible: TN1 Resistant: YHY15		The abundance and expression level of circRNAs in YHY15 BPHs were higher than those in TN1, and 19 circRNAs have been identified as possibly involved in the autophagy process.	[61]
	Susceptible: TN1 Resistant: YHY15		The 26 miRNAs showed significantly differential expressions between two libraries. Moreover, it also determined that a majority of differential miRNAs were involved in the ’Metabolism’ pathway.	[62]
Proteomics	Susceptible: TN1 Resistant: YHY15		The 151 upregulated DEPs, which were involved in apoptosis metabolism, response to chemicals, response to oxygen-containing compounds, and regulation of the response to stress might be related in BPHs’ adaptation to rice resistance.	[63]
Metabolomics	Susceptible: TN1 Resistant: YHY15		The levels of most amino acids in honeydew decreased, and the levels of succinic acid and malic acid were elevated in the BPHs of YHY15 compared with TN1.	[64]
	Susceptible: TN1	Susceptible: TN1 Resistant: YHY15	BPHs feeding on resistant plants had lower levels of amino acids, glucose, fatty acids, and TCA cycle intermediates than on the susceptible ones. The levels of these metabolites recovered after 24 h of feeding and were accompanied with increased levels of trehalose, choline metabolites, and nucleosides/nucleotides.	[65]
	Susceptible: Huang Huazhan	Susceptible: TN1 Resistant: IR56	BPHs feeding on IR56 plants exhibited significant decreases in concentrations of most of the detected sugars, vitamins, and some essential amino acids, but higher levels of most amides, free fatty acids, and some non-essential amino acids.	[66]
	Susceptible: TN1 Resistant: IR36	Resistant: IR36	Alanine was one of the key biomarkers of BPH adaptation to the resistant rice variety IR36. Alanine aminotransferase (ALT)-mediated alanine transfer to pyruvate was necessary and sufficient for the adaptation.	[67]
	Susceptible: TN1	Susceptible: 9311 Resistant: NIL-*Bph6*, NIL-*Bph9*	Insects that fed on resistant rice transformed triglyceride (TG) to phosphatidyl choline (PC) and digalactosyl diacylglycerol (DGDG), with these lipid classes showing significant alterations in fatty acid composition.	[68]
Microbiomics	Susceptible: TN1 Resistant: Mudgo, ASD7, IR42, and IR56		The composition of symbiotic bacteria within populations of different harmful brown planthopper species varies were evident, with ASD7 and Mudgo-resistant varieties having more symbiotic bacteria than the susceptible variety TN1.	[69]
	Susceptible: TN1 Resistant: ASD7		Comparative analysis showed that significant differences in the profile of gut bacterial communities existed between the two BPH populations. We found the relative abundances of two subdominant phyla and two subdominant classes were significantly different.	[70]

## Data Availability

No data were used for the research described in this article.
